# Association between prenatal opioid exposure and health, education, and foster care between ages 0 and 18

**DOI:** 10.1093/pnasnexus/pgag024

**Published:** 2026-03-03

**Authors:** Gaëlle Simard-Duplain, Jonathan Zhang

**Affiliations:** Department of Economics, Carleton University, Ottawa, ON, Canada K1S 5B6; Sanford School of Public Policy, Duke University, Durham, NC 27708, USA; National Bureau of Economic Research, Cambridge, MA 02138, USA

**Keywords:** opioid crisis, intergenerational impacts

## Abstract

The opioid crisis has emerged as a critical public health challenge, yet the long-term outcomes of children exposed before birth remain underexamined. Prior studies have focused on acute neonatal outcomes or adult opioid misuse, leaving the broader developmental outcomes largely unexplored. Here, we show that children in British Columbia with prenatal opioid exposure—identified through comprehensive linked administrative data—face significant and enduring deficits in health, education, and social well-being that extend into adolescence. These findings reveal associations that go beyond clinically diagnosed neonatal abstinence syndrome, highlighting the importance of addressing even lower levels of prenatal exposure. Propensity score matching qualitatively corroborates these findings. By examining multiple levels of prenatal exposure and linking them to a wide array of child outcomes, our work underscores the urgent need for enhanced prenatal screening, targeted interventions, and integrated policymaking to break the intergenerational cycle of opioid-related harm.

Significance Statement Although much attention has been given to rising opioid fatalities among adults, less is known about the enduring consequences for children exposed to opioids before birth. By examining nearly two decades of linked administrative data in British Columbia, this study reveals that in utero opioid exposures occur at rates far exceeding those suggested by neonatal abstinence syndrome diagnoses. Moreover, such exposures correspond to notable and lasting deficits in child health, human capital, and well-being. These findings underscore the urgent need for preventative strategies, early interventions, and public policies that address prenatal opioid use to mitigate its intergenerational consequences.

## Introduction

The opioid crisis has reached unprecedented levels in the United States and Canada (1, 2). Between 2002 and 2022, the age-adjusted rate of drug overdose deaths rose from 8.2 to 32.6 per 100,000 in the United States (3). In Canada, the rate has risen from 7.8 in 2016 to 21.3 in 2023 (4).

Much of the research on the opioid crisis has focused on those directly impacted by drug use and the middle-aged or elderly population (5, 6). One crucial dimension that has largely been ignored is the crisis’ impact on the next generation, sometimes referred to as a “hidden epidemic” (7). Neonatal abstinence syndrome (NAS)—a clinically evident withdrawal condition affecting approximately six out of every 1,000 US births (8)—has drawn attention, with prior work documenting adverse newborn outcomes (9–11) and, in smaller cohorts, early childhood neurodevelopmental delays (12–16) and worse test scores (17). However, data limitations have hindered a more expansive assessment of differences in outcomes for children exposed to opioids in utero beyond the newborn period (7, 18). While these differences do not necessarily capture the causal effect of in utero exposure on children, they inform compounded vulnerabilities faced by exposed children.

In this study, we overcome these data barriers by drawing on more than two decades of comprehensive administrative records from British Columbia (BC), Canada. This province of over 5.7 million residents currently has a drug overdose rate of 45.7 per 100,000, exceeding the US national average. Crucially, our linked data enable us to construct different measures (relating to severities) of potential in utero opioid exposure—ranging from a clinical NAS diagnosis to broader indicators of maternal opioid use—and follow children from birth to adolescence. In addition, we leverage data sources from a variety of BC ministries and programs, which allows us to examine children's outcomes in a wide range of domains. We also capture and control for rich sociodemographic maternal characteristics, which may influence child outcomes. We therefore provide a unified analysis of the associations between prenatal opioid exposure and healthcare utilization, educational outcomes, inclusive education services (often colloquially known as “special needs” services), interactions with child protective services, and government welfare usage. By capturing a breadth of outcomes and stratifying exposure severity, our study offers the most comprehensive assessment of how the opioid crisis may extend to subsequent generations. It is important to note that our analysis identifies correlations between prenatal opioid exposure and subsequent child outcomes, rather than establishing a causal relationship.

## Materials and methods

We analyzed administrative data from the Canadian province of BC, obtained through BC's Data Innovation Program (DIP). The DIP is a comprehensive, multisectoral linkage platform that integrates health, education, and social service records for BC residents and their families. Its unique individual and family identifiers enable the tracking of individuals across multiple domains, including births and vital statistics (Perinatal Data Registry, BC Vital Events and Statistics for Births and Deaths), education (K–12 student assessments and enrollment), health (Discharge Abstract Database, National Ambulatory Care Reporting System, Medical Services Plan, PharmaNet), and government and child welfare services (BC Employment and Assistance, Child Welfare Program). The DIP has been used extensively to investigate the determinants of health, well-being, and development. Except for emergency department data, which we observed with some uncertainty before 2014, all datasets span the 2000–2020 period, offering a rich, longitudinal perspective on maternal and child outcomes. The Supplementary material explains the data in more detail.

We used the Perinatal Data Registry to identify all live births in BC from 2000 April 1 to 2020 January 1. This includes 897,668 births (our sample size) occurring in BC hospitals as well as home births attended by registered midwives, thus capturing the full spectrum of births in the province. It provides detailed information on birth outcomes and on maternal and newborn episodes of care from the antenatal through postpartum period. These records were then linked to the additional administrative datasets described above, enabling comprehensive follow-up of both mothers and children.

### Measures of in utero opioid exposure

We identified potential in utero opioid exposure using three measures, ranked by plausibly decreasing severity of exposure (“dose”). First, we used a diagnosis of NAS at the time of birth, capturing the most clinically evident presentations of opioid withdrawal (“NAS-diagnosed”). Second, we leveraged broader administrative health records beyond the delivery episode, encompassing any infant diagnosed with NAS or with “affected by maternal use of drugs of addiction” in the 60 days following birth, as well as any mother diagnosed with drug abuse or dependence during pregnancy (“Maternal drug abuse”). This measure accounts for underdiagnoses or discrepancies in NAS reporting within the hospital setting (19). Third, we included mothers who screened or tested positive for drug abuse at childbirth or who filled at least three opioid prescriptions during pregnancy (“Positive screen/multiple opioid prescriptions”). While prescription fills do not guarantee consumption, multiple fills over pregnancy plausibly indicate sustained maternal opioid use. Because our data are constrained by the detail present in the ICD diagnosis (both ICD-9 and ICD-10), we could not completely separate opioids from other potentially misused substances; however, opioids were the most commonly abused drug during this period (20). Additional details on these classifications can be found in the Supplementary material.

### Outcomes

We capture outcomes in three broad domains—health, education, and social welfare—annually, spanning birth through the child's 18th year. In the health domain, we construct measures of total medical spending and prescription drug spending from administrative records, reflecting government expenditures for medical spending and total government and individual out-of-pocket costs for prescription drug spending.

In the education domain, we examine both inclusive education designations (which in our setting refer to students with disabilities or diverse abilities) and academic performance. Inclusive education designations are derived from school enrollment records for funding purposes, which categorize specific disabilities (e.g. physical and chronic impairments, mental health or behavioral support needs, intellectual disabilities and autism spectrum disorder, learning disabilities, and sensory impairments). We also measure standardized test scores in Grades 4 and 7 and high-school grade point average (GPA) in SDs, an indicator for high-school graduation by age 18, and a proxy for grade retention (whether a student's grade placement is behind the expected level for their age).

Lastly, we investigate social well-being through indicators of government welfare usage and child welfare involvement. We construct annual income assistance and disability benefits totals for the child and mother since payments are typically issued to the mother. We further identify whether a child has ever been under the supervision of child protective services or placed in foster care.

### Statistical analysis

We examine the relationship between in utero opioid exposure and child outcomes by estimating the following model for child *i* at age *t*:


(1) $$Y_{i}^{t}=\;\beta \;\mathrm{Exposur}e_{i}\;+\;\lambda X_{i}\;+\epsilon_{i}\text{■}$$

where $\mathrm{Exposur}e_{i}$ indicates whether child *i* experienced one of our three measures of potential in utero opioid exposure, and *X_i_* is a vector of birth and maternal characteristics: fixed effects for birth year, birth month, mother's age at birth, parity, three-digit postal code, and calendar year, as well as mother's world region of birth, the mother's marital status from birth records, an indicator for receiving government benefits during pregnancy, and categorical bins for levels of low income. Because Indigenous people in Canada face the ongoing repercussions of colonialism and discrimination in many domains, we also control for a variable that captures the child's Indigenous ancestry (self-reported in school enrollment records) and band membership (as recorded in their health insurance plan). For simplicity, we refer to this variable as “Indigenous identity” from here on. These variables are explained in detail in the Supplementary material.

We run separate regressions for each of the three exposure measures, comparing these exposed groups to children with no indication of in utero opioid exposure on any measure. We also estimate the regressions separately by age (from 1 to 18), which effectively allows the impacts of controls to vary flexibly with age. The parameter of interest is *β*, which captures the average difference in child outcome at a given age for those with a particular exposure relative to children with no exposure. We report βand two-sided 95% CIs in graphical and table format.

In a sensitivity analysis, we estimate a propensity score matching approach to ensure common support in maternal covariates. The details of the propensity score matching analysis can be found in the Supplementary material.

While sibling fixed-effects designs can strengthen causal inference, they substantially restrict the analytic sample to a small subset of families with discordant exposure and exclude many children from highly affected households. Our goal here is to characterize population-level associations across all births in the province, and thus we prioritize external validity and comprehensive coverage.

## Results

We begin by examining the maternal and birth characteristics associated with each of the three exposure measures. Figure S1 presents coefficients from a multivariate regression scaled by the baseline mean (probability) of each measure. Mothers of exposed newborns are, on average, younger, unmarried or of unknown marital status, born in Canada or the United States, and have lower incomes—including reliance on government benefits during pregnancy. This analysis also informs about the differences across the three groups of mothers of exposed newborns. Although the analyses that follow control for these covariates, it is important to bear these selection patterns in mind when interpreting the results. We return to this issue in the Discussion section.

**Fig. 1.**
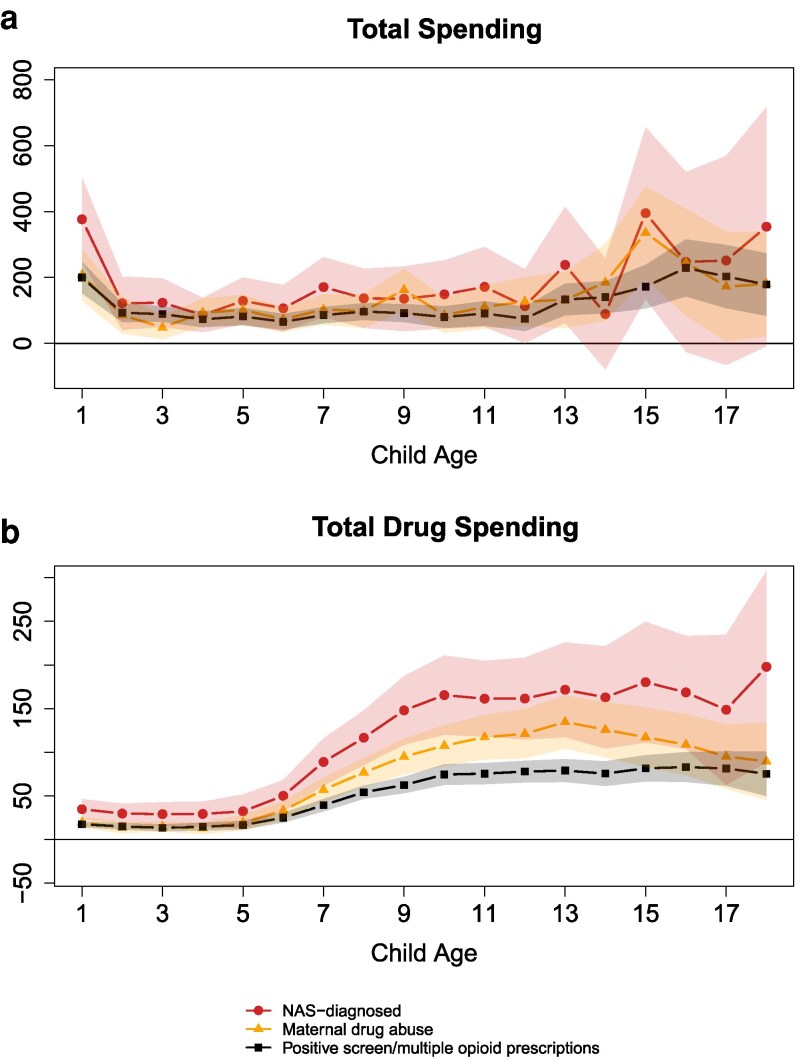
Association between healthcare utilization and in utero opioid exposure, relative to nonexposed, ages 1–18. Each point is an estimated coefficient on the exposure measure (representing the difference between exposed and nonexposed), along with its 95% CI, from Eq. 1: linear regression comparing the stated outcome variable between the exposed and those never exposed, controlling for birth year, birth month, mother's age-fixed effect, parity, three-digit postal code, and calendar-year-fixed effects. The means for nonexposed children at ages 1–5, 6–12, and 13–18 for (a) total spending are $482, $287, and $467 and (b) drug spending are $48, $83, and $166. All dollar amounts are in real 2021 Canadian dollars.

Figure 1 presents the gap in total healthcare spending (panel a) and prescription drug spending (panel b) between children exposed to opioids in utero and those with no exposure, from ages 1 to 18. In panel (a), children diagnosed with NAS incur an average of $376 more in healthcare expenditures in the first year of life, an 80% increase over that of nonexposed children—and maintain steadily higher costs thereafter. Notably, children who meet either of the two less severe exposure criteria also experience significant and persistent spending increases, averaging ∼70–80% of the NAS figure over the 18 years.

In panel (b), prescription drug spending exhibits an upward trend. Between ages 1 and 5, NAS-diagnosed children spend roughly two-thirds more on prescriptions than their nonexposed counterparts. Between ages 13–18, they spend an additional $149–198 annually, over a mean of $166 for the nonexposed group. Even children with potential in utero exposure but no formal NAS diagnosis show statistically and economically meaningful increases in prescription costs, underscoring the broader healthcare burden associated with prenatal opioid exposure. Children in the positive screen/multiple opioid prescriptions group have 40–50% of total drug spending compared with the NAS diagnosed; the maternal drug abuse group falls in the middle. Figure S2 shows associations with emergency department and hospital visits that are generally not statistically significant, potentially reflecting the infrequency of these occurrences.

**Fig. 2.**
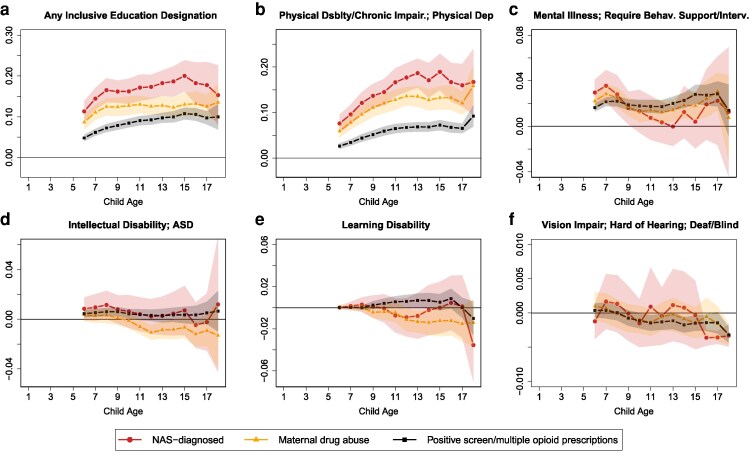
Association between inclusive education designations and in utero opioid exposure, relative to nonexposed, ages 1–18. Each point is an estimated coefficient on the exposure measure (representing the difference between exposed and nonexposed), along with its 95% CI, from Eq. 1: linear regression comparing the stated outcome variable between the exposed and those never exposed, controlling for fixed effects for birth year, birth month, mother's age at birth, parity, three-digit postal code, calendar year, mother's world region of birth, marital status, Indigenous identity, an indicator for receiving government benefits during pregnancy, and categorical bins for low-income proxy. The means for nonexposed children at ages 6–12 and 13–18 for (a) any inclusive education designations: 0.077, 0.144; (b) physical: 0.014, 0.016; (c) behavioral and mental: 0.019, 0.032; (d) intellectual disability, ASD: 0.021, 0.030; (e) learning disability: 0.021, 0.062; (f) vision, hearing: 0.003, 0.003.

A possible explanation for the elevated healthcare utilization among exposed children is their increased likelihood of disability and healthcare needs. As shown in Fig. 2, the probability of receiving any inclusive education designation by school entry is 11.3 percentage points (pp) higher for children diagnosed with NAS and 4.8 pp higher for those meeting our third, most expansive exposure measure (positive screen/multiple opioid prescriptions), relative to nonexposed children. These differences increase markedly over time, reaching a 20.0 pp gap for NAS-diagnosed children and 10.8 pp for the third measure by adolescence—effectively doubling the inclusive education rate compared with unexposed peers.

As we break down the types of inclusive education designations, the results become somewhat noisy, especially for the NAS diagnosed; however, some clear patterns emerge. Most of the increases in inclusive education designations come from increases in physical disabilities and chronic impairments, although mental illness also rises meaningfully. Notably, the gap in physical disabilities (panel b) is largest among the NAS-diagnosed group, likely reflecting the heightened clinical scrutiny at birth for infants with evident physical defects (20). In contrast, elevated rates of mental and learning-related disabilities are largely similar across most of the exposure measures (panels c and e), underscoring the broad and persistent developmental challenges associated with prenatal opioid exposure. It is worth noting that the size of the CIs reflects the prevalence of each measure, and these are competing designations, with children typically only receiving one in the school records.

**Fig. 3.**
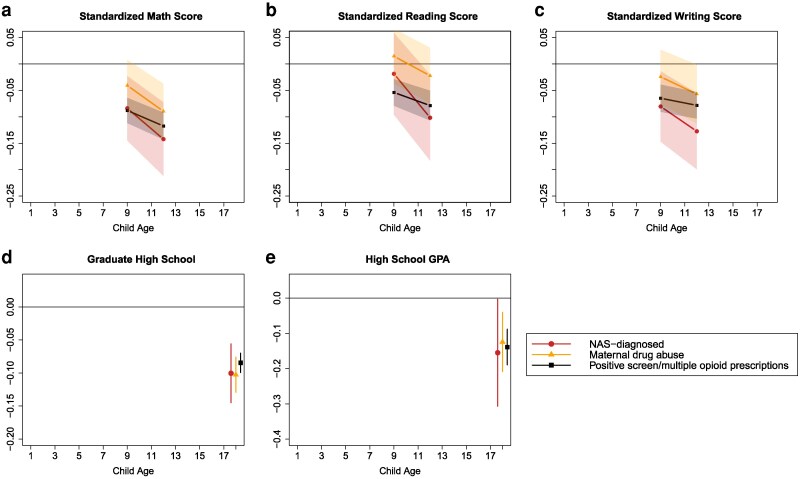
Association between educational outcomes and in utero opioid exposure, relative to nonexposed, ages 1–18. Each point is an estimated coefficient on the exposure measure (representing the difference between exposed and nonexposed), along with its 95% CI, from Eq. 1: linear regression comparing the stated outcome variable between the exposed and those never exposed, controlling for fixed effects for birth year, birth month, mother's age at birth, parity, three-digit postal code, calendar year, mother's world region of birth, marital status, Indigenous identity, an indicator for receiving government benefits during pregnancy, and categorical bins for low-income proxy. The means for nonexposed children for standardized test scores in (a) math, (b) reading, and (c) writing (grades 4 and 7 average): 0.015, 0.110, and 0.182; (d) graduating high school: 0.725; (e) high school GPA: 3.229.

Figure 3 shows NAS-diagnosed children score 2–8% of an SD lower than nonexposed peers in mathematics, reading, and writing in grade 4 (panels a–c), and the other two exposure groups show similar deficits. Shortfalls in math scores are the largest. By grade 7, these academic gaps widen by ∼5–10% of an SD across all three subjects. Note that for maternal drug abuse, the associations with test scores are generally not statistically significant. Because exposed children are also less likely to participate in standardized testing—perhaps due to higher rates of inclusive education designations—these estimates likely understate the true extent of their academic deficits. Beyond test scores, exposed children are 1–2 pp more likely to be held back a grade (Fig. S3) and 8–10 pp less likely to graduate high school on time (Fig. 3d). Panel (e) shows that their high-school GPAs are likewise lower by 12–16% of an SD. Due to wide CIs, there do not appear to be statistically significant differences in educational outcomes across the three different exposure measures.

**Fig. 4.**
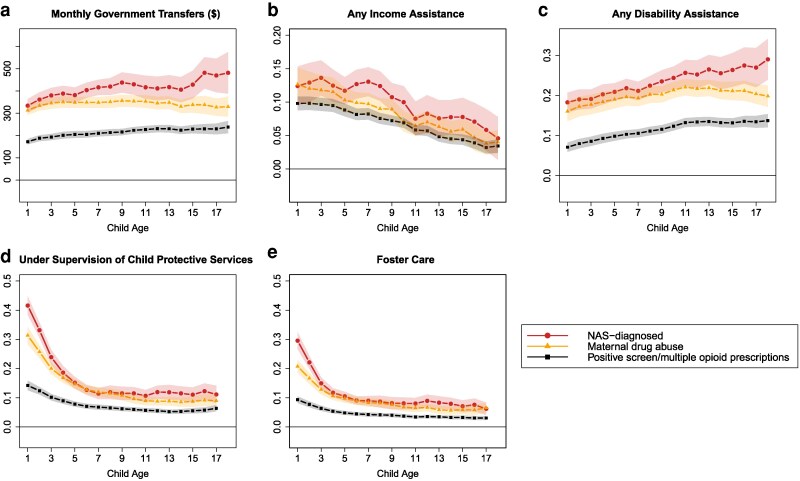
Association between government welfare utilization and child welfare and in utero opioid exposure, relative to nonexposed, ages 1–18. Each point is an estimated coefficient on the exposure measure (representing the difference between exposed and nonexposed), along with its 95% CI, from Eq. 1: linear regression comparing the stated outcome variable between the exposed and those never exposed, controlling for fixed effects for birth year, birth month, mother's age at birth, parity, three-digit postal code, calendar year, mother's world region of birth, marital status, Indigenous identity, an indicator for receiving government benefits during pregnancy, and categorical bins for low-income proxy. The means for nonexposed children at ages 1–5, 6–12, and 13–18 for (a) monthly benefits: $59, $57, and $62; (b) any welfare: 0.051, 0.035, and 0.024; (c) any disability benefits: 0.012, 0.018, and 0.026; (d) under CPS supervision: 0.008, 0.009, and 0.009; (e) under foster care: 0.004, 0.004, and 0.004. All dollar amounts are in real 2021 Canadian dollars.

Families of children exposed to opioids in utero also exhibit markedly higher reliance on government welfare programs. As shown in Fig. 4a, total monthly government transfers for families of NAS-diagnosed children are consistently $333–$482 above the $60 average for nonexposed children—initially dominated by income assistance but increasingly supplemented by disability assistance in later years. Families of children meeting the maternal drug abuse criterion receive similar total government transfers as NAS-diagnosed children until age 5. From ages 6–18, their transfers are ∼80% as large as the associations estimated for NAS-diagnosed children. Families of children meeting the positive screen/multiple opioid prescription criterion receive $172–$238 per month more than nonexposed children (∼50% the NAS-diagnosed estimate). Child welfare involvement is similarly elevated. NAS-diagnosed, maternal drug abuse, and positive screen/multiple opioid prescription groups are 42, 32, and 14 pp more likely, respectively, to come under the supervision of child protective services in the first year (versus 0.8 pp for nonexposed children; panel d). Approximately two-thirds of these cases involve foster care (panel e). Although this heightened risk tapers by school age, it remains significantly above baseline throughout adolescence.

**Fig. 5.**
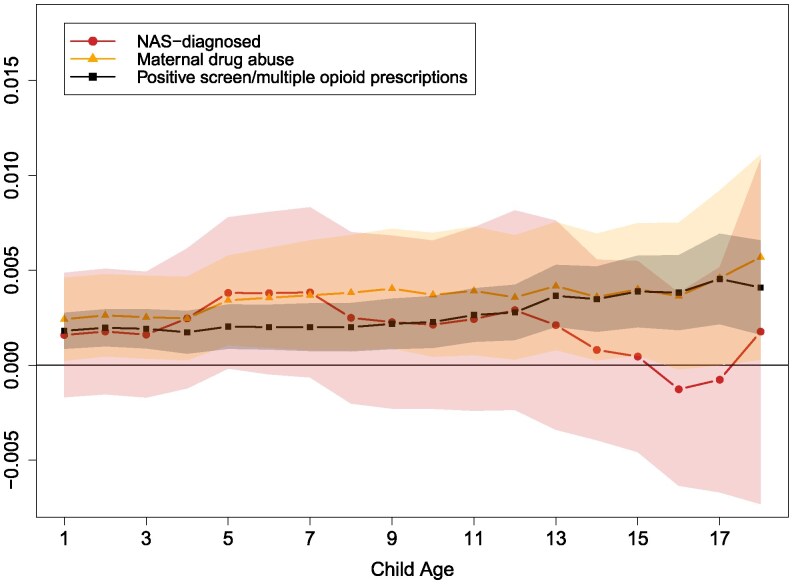
Association between cumulative mortality and in utero opioid exposure, relative to nonexposed, ages 1–18. Each point is an estimated coefficient on the exposure measure (representing the difference between exposed and nonexposed), along with its 95% CI, from Eq. 1: linear regression comparing the stated outcome variable between the exposed and those never exposed, controlling for fixed effects for birth year, birth month, mother's age at birth, parity, three-digit postal code, calendar year, mother's world region of birth, marital status, Indigenous identity, an indicator for receiving government benefits during pregnancy, and categorical bins for low-income proxy. The mean (cumulative) mortality for nonexposed children at ages 1–5, 6–12, and 13–18 are 0.002, 0.003, and 0.003, respectively.

Finally, Fig. 5 investigates cumulative mortality between ages 1 and 18. Maternal drug abuse and positive screen/multiple opioid prescriptions is associated with 0.2–0.5 pp higher cumulative mortality compared with the nonexposed baseline of 0.2–0.3 pp. The differences between NAS-diagnosed and nonexposed is not statistically significant; however, the three exposure measures appear to carry similarly elevated risks. The CIs are wide; however, it appears that the elevated mortality risk rises with age for children exposed to maternal drug abuse and positive screen/multiple opioid prescriptions.

Consistent with the pattern observed across outcomes, these findings suggest a gradient: more severe exposure measures are likely to reflect greater in utero opioid exposure “dose,” and consequently, be associated with worse outcomes. Yearly regression coefficients and standard errors are reported in Tables S1–S5.

**Fig. 6.**
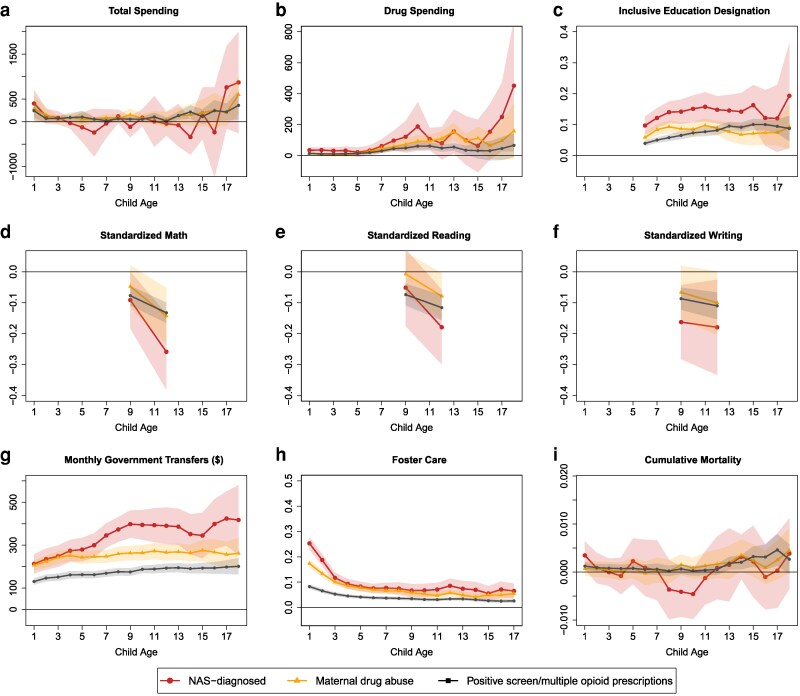
Propensity score matching: association between main outcomes and in utero opioid exposure, relative to nonexposed, ages 1–18. Each point is an estimated coefficient on the exposure measure (representing the difference between exposed and nonexposed), along with its 95% CI, for a one-to-one nearest neighbor propensity score-matched sample. The regressions control for three-digit postal code; (a-i) all other covariates are well-balanced (extended Fig. 4). See Supplementary material for more details on the propensity score-matching procedure.

We also performed a sensitivity analysis using one-to-one propensity score matching to pair each child in the three exposed groups with a comparable nonexposed child. This approach improves comparability by selecting nonexposed children with similar pre-birth characteristics. As shown in Fig. S4, the matching procedure achieves good covariate balance (i.e. exposed and nonexposed children share common support) across a wide range of maternal characteristics prior to birth. Figure 6 presents estimated differences between exposed and nonexposed children from ages 1 to 18 across nine key outcomes. The results from the matched sample are qualitatively—and often quantitatively—consistent with our main estimates. Estimates for healthcare utilization are noisier, particularly for the smaller NAS-diagnosed group, but the magnitudes and the increase in spending with age mirror our primary findings. Inclusive education designations follow comparable patterns. The deficits in standardized test scores are somewhat larger after matching: for example, grade 7 scores for NAS-diagnosed children are 0.20–0.25 of an SD lower than in the unmatched estimates. Government transfers and foster care outcomes remain nearly identical in both magnitude and trajectory. As before, cumulative mortality estimates are imprecise and statistically significant only for the positive screen/multiple prescriptions group, primarily after age 13.

## Discussion

The opioid crisis stands as one of the most pressing public health emergencies in North America, yet the implications for children born into disadvantaged circumstances and their potential policy solutions remain insufficiently explored (7, 18, 21, 22). Drawing on two decades of comprehensive administrative data, this study underscores the significant and lasting associations of prenatal opioid exposure with children's health, educational attainment, and well-being from birth through adolescence. While these associations may or may not be causal, they highlight the compounded difficulties exposed children face throughout their lifetime.

Consistent with prior findings (19, 23–25), we find that the prevalence of potential in utero exposure far exceeds what would be captured by NAS diagnoses alone. Even excluding exposure implied by opioid prescriptions, ∼874 newborns in BC per year show indicators of exposure. Extrapolating this undercounting rate to the United States—i.e. assuming the ratio of NAS diagnosis to potential exposure is constant across BC and the United States—suggests that in 2023, ∼95,000 American infants may have been exposed to opioids in utero—on par with the yearly number of overdose fatalities (see Supplementary material for calculations). Moreover, even lower levels of exposure are associated with persistent and substantial adverse outcomes, highlighting the need to broaden research and intervention efforts beyond clinically evident NAS, which may only capture the most severe or detectable cases at birth (19).

Several mechanisms may underpin these intergenerational associations. Opioids can directly disrupt fetal neurodevelopment, potentially leading to cognitive, behavioral, and emotional challenges in childhood (18, 26–29). Additionally, maternal and household contexts which may themselves contribute to or be associated with opioid use during pregnancy—encompassing parenting capacity, socioeconomic status, household conditions and resources, and interactions with social services—likely magnify or sustain these developmental risks (29–32). Importantly, controlling for maternal socioeconomic indicators does not eliminate the observed deficits, suggesting that prenatal opioid exposure itself, rather than just household disadvantage, plays a key role.

The associations we document are broadly consistent with prior research that causally links opioid exposure—typically not limited to in utero exposure—to adverse child outcomes. Studies exploiting variation in local opioid supply, such as that induced by Purdue Pharma's marketing practices, find that greater exposure lowers standardized test scores and high school exit examination pass rates and increases the likelihood that a child lives apart from a parent (33, 34). Evidence on the impact of supply-side policies, such as prescription drug monitoring programs, is mixed: some studies report reductions in child removals (35), while others find increases in child maltreatment (36). Work focusing specifically on prenatal opioid exposure among Medicaid births in Wisconsin documents poorer newborn health, which in turn predicts worse outcomes later in childhood (37). Our study extends this literature by directly measuring children's health and educational outcomes rather than inferring effects from birth outcomes alone.

Our findings also align with a broader literature on prenatal exposure to other substances and early-life shocks. Prenatal alcohol exposure has been shown to reduce educational attainment, cognitive and noncognitive skills, and later labor market earnings (38, 39). Similarly, maternal stress during pregnancy is linked to lower birth weight and modest but lasting impacts on health. The positive association we find between in utero opioid exposure and later medication use is consistent with this evidence, mirroring findings that children exposed to severe prenatal stress—such as the death of a family member—exhibit increased use of mental health medications (40, 41).

Our findings have important policy implications. First, earlier prenatal screening and supportive and compassionate interventions for at-risk mothers could help mitigate harm for both mothers and children (42–44). Second, the interconnected deficits uncovered by our linked administrative data also point to the importance of comprehensive, integrated services across health, child welfare, and education systems to address the broad and interconnected needs of families. Recent nonpharmacological approaches have shown promise for treating infants with NAS (45, 46), and medication-assisted therapy for opioid use disorder during pregnancy appears to confer benefits to child outcomes (47–49). Determining whether such interventions can improve outcomes into adolescence and beyond remains an important question (50). Finally, researchers and policymakers need to put children and families at the forefront of discussions around opioid policies.

Despite these contributions, the study has limitations. Observational analyses are subject to unobserved confounders and selection bias, and our results should not be interpreted as causal. For instance, complex and changing household and environmental circumstances may confound the association between in utero exposure and child outcomes (21, 33, 51). Moreover, socioeconomic determinants of health could simultaneously shape maternal opioid use and child outcomes (52, 53). Reliance on ICD coding may under- or overestimate true exposure rates. Opioid and drug use is incompletely measured. Missing data for children who relocate outside of BC, are homeschooled, or seldom use healthcare services also restricts outcome measurement. Finally, because we cannot observe the newest birth cohorts through adolescence, our findings may not fully capture the latest trajectories of children born amid recent developments in the opioid crisis.

We hope that our study encourages future research into the causal effects of in utero opioid exposure on children and interventions that can mitigate adverse outcomes for children exposed to opioids in utero. While our understanding of effective policies to improve general child outcomes is substantial, it remains unclear whether these same policies benefit opioid-exposed newborns or whether tailored prenatal and postnatal supports are more effective (54). By combining large-scale data linkage with evidence-based practices, future work can help break the cycle of disadvantage that threatens to perpetuate the impact of the opioid crisis across multiple generations.

## Supplementary Material

pgag024_Supplementary_Data

## Data Availability

The data analyzed in this paper comes from British Columbia's Data Innovation Program. Information about data access can be found on Population BC's website at https://www.popdata.bc.ca/projects/data-innovation-program. For those who are interested, we will do our best to assist them in gaining access to the data. All programming code and replication instructions can be found at: https://github.com/jzhang722/prenatal-opioid/.

## Disclaimer

The following material was developed as part of a project titled “Family and intergenerational impacts of poverty and income assistance” (University of British Columbia, University of Toronto, Carleton University, McMaster University), Province of British Columbia. The following data sets were used: BC Employment and Assistance (BCEA), Registration and Premium Billing, Discharge Abstract Database (Hospital Separations), Medical Services Plan (MSP) Payment Information File, Consolidation File, BC Vital Events and Statistics (Births and Deaths), Perinatal Data Registry, Pharmanet, National Ambulatory Care Reporting System (NACRS) Program, K to 12 Student Demographics and Achievements, and Linked data files from the Ministry of Child and Family Development, and Corrections—Adult Community and Custody. Further information regarding these data sets can be found at https://catalogue.data.gov.bc.ca/organization/data-innovation-program-dip. All inferences, opinions, and conclusions in these materials are those of the authors. They do not reflect the opinions or policies of the provider(s) of the data upon which they are based.
