# Supplementary material for: Association between prenatal opioid exposure and health, education, and foster care between ages 0 and 18
Source: PNAS Nexus. 2026 Mar 3;5(3):pgag024. doi: 10.1093/pnasnexus/pgag024 (PMC12954679; doi:10.1093/pnasnexus/pgag024)
Supplement: pgag024_Supplementary_Data [file pgag024_supplementary_data.pdf]

Supplemental Materials for
"Association Between Prenatal Opioid Exposure and Health, Education, and Foster Care Between Ages 0-18"

Gaëlle Simard-Duplain and Jonathan Zhang

Table of Contents

A. Supplemental Figures and Tables
B. Definitions
C. Propensity Score Matching
D. Calculation of the Number of Potentially In Utero Exposed Newborns

A. Supplemental Figures and Tables

Supplemental Figure 1: Associations between exposure measures and birth and maternal characteristics

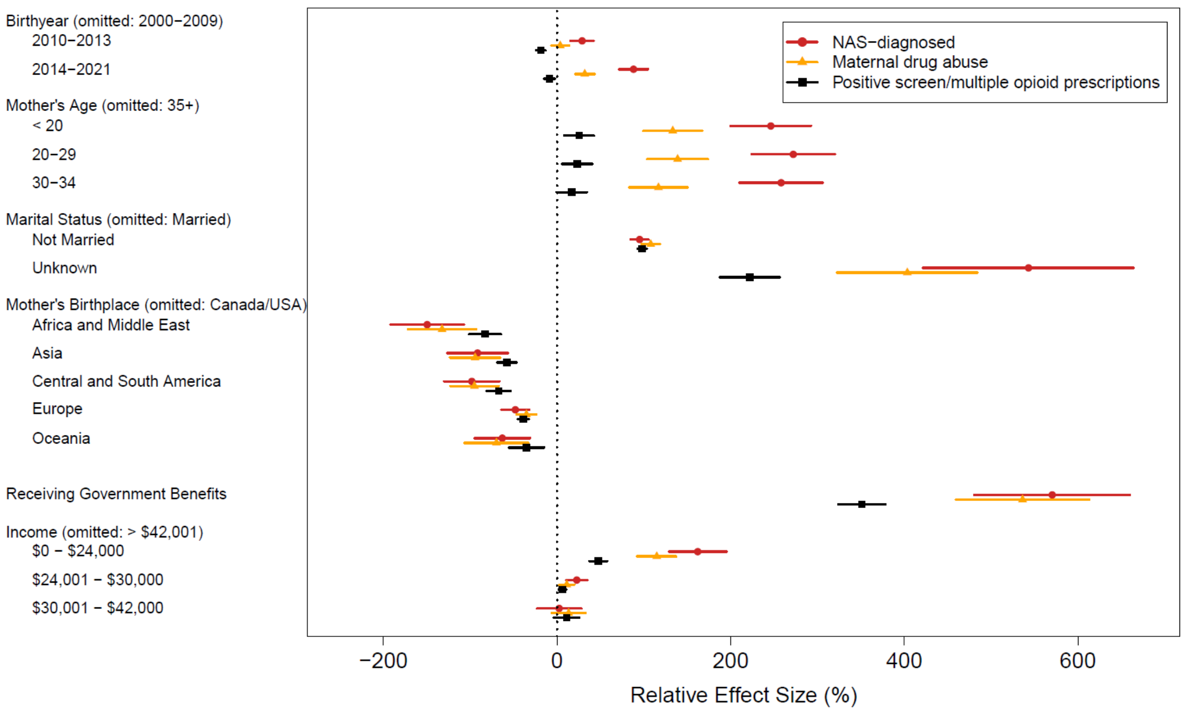

Each point is a scaled regression coefficient from a multivariate regression of an indicator for the exposure measure on birth and maternal characteristics at birth. Three regressions are run for each exposure measure, on the sample of all newborns in BC. The regression coefficients are scaled by the baseline mean of the outcome variable (i.e., the mean fraction of newborns in BC with each exposure measure). 95% confidence intervals and omitted categorical variables are also displayed in the figure.

Supplemental Figure 2: Association between emergency department (ED) and hospital visits and in utero opioid exposure, relative to non-exposed, ages 1-18

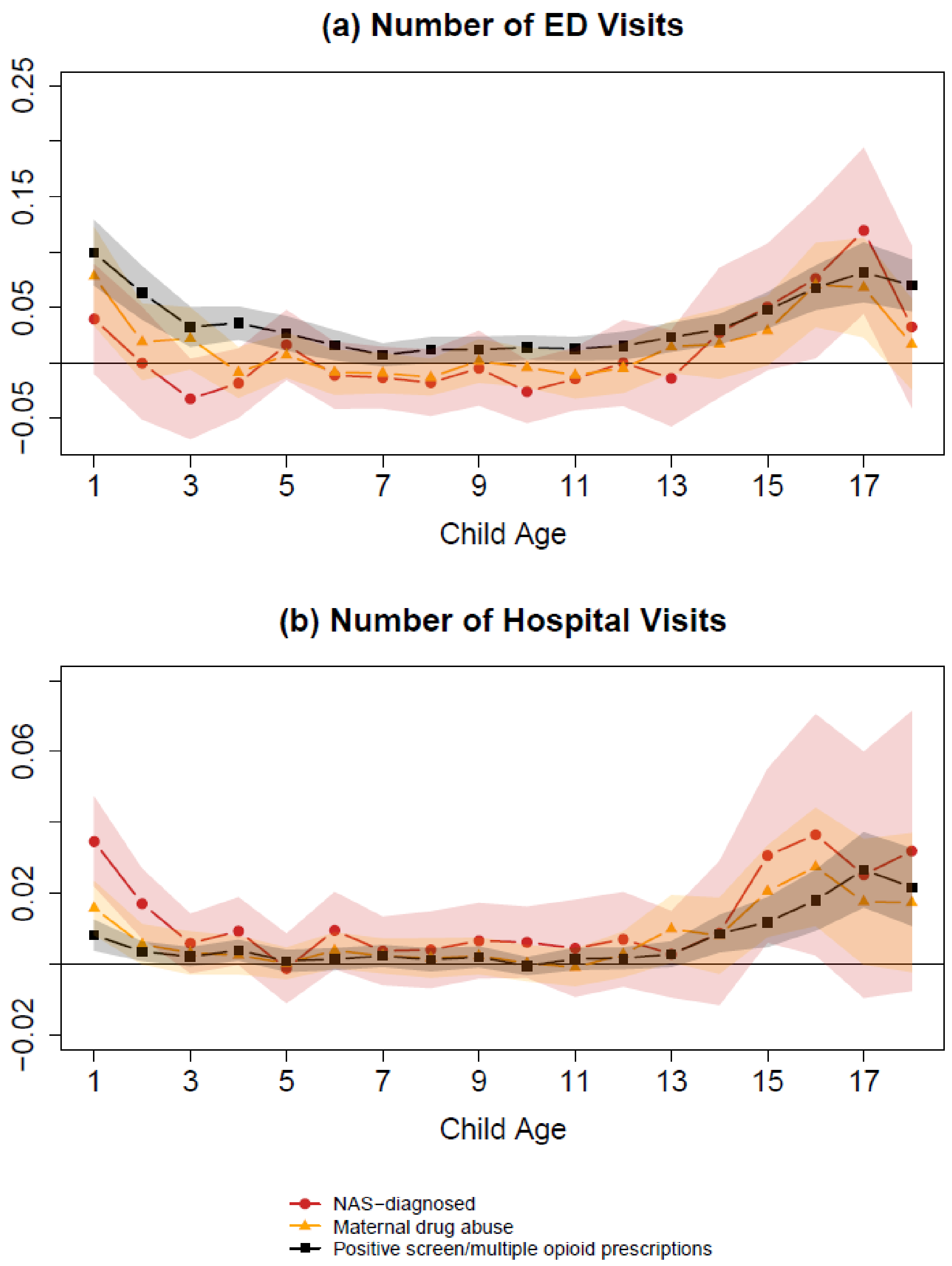

Each point is an estimated coefficient on the exposure measure (representing the difference between exposed vs non-exposed), along with its 95% confidence interval, from equation 1: linear regression comparing the stated outcome variable between the exposed and those never exposed, controlling for fixed effects for birth year, birth month, mother's age at birth, parity, 3-digit postal code, calendar year, mother's world region of birth, marital status, Indigenous identity, an indicator for receiving government benefit during pregnancy, and categorical bins for low income proxy. Means for non-exposed children at ages 1-5, 6-12, and 13-18 for ED visits: 0.32, 0.15, 0.19; and hospital admissions: 0.015, 0.014, 0.030.

Supplemental Figure 3. Association between writing any standardized test and grade retention, and in utero opioid exposure, relative to non-exposed, ages 1-18

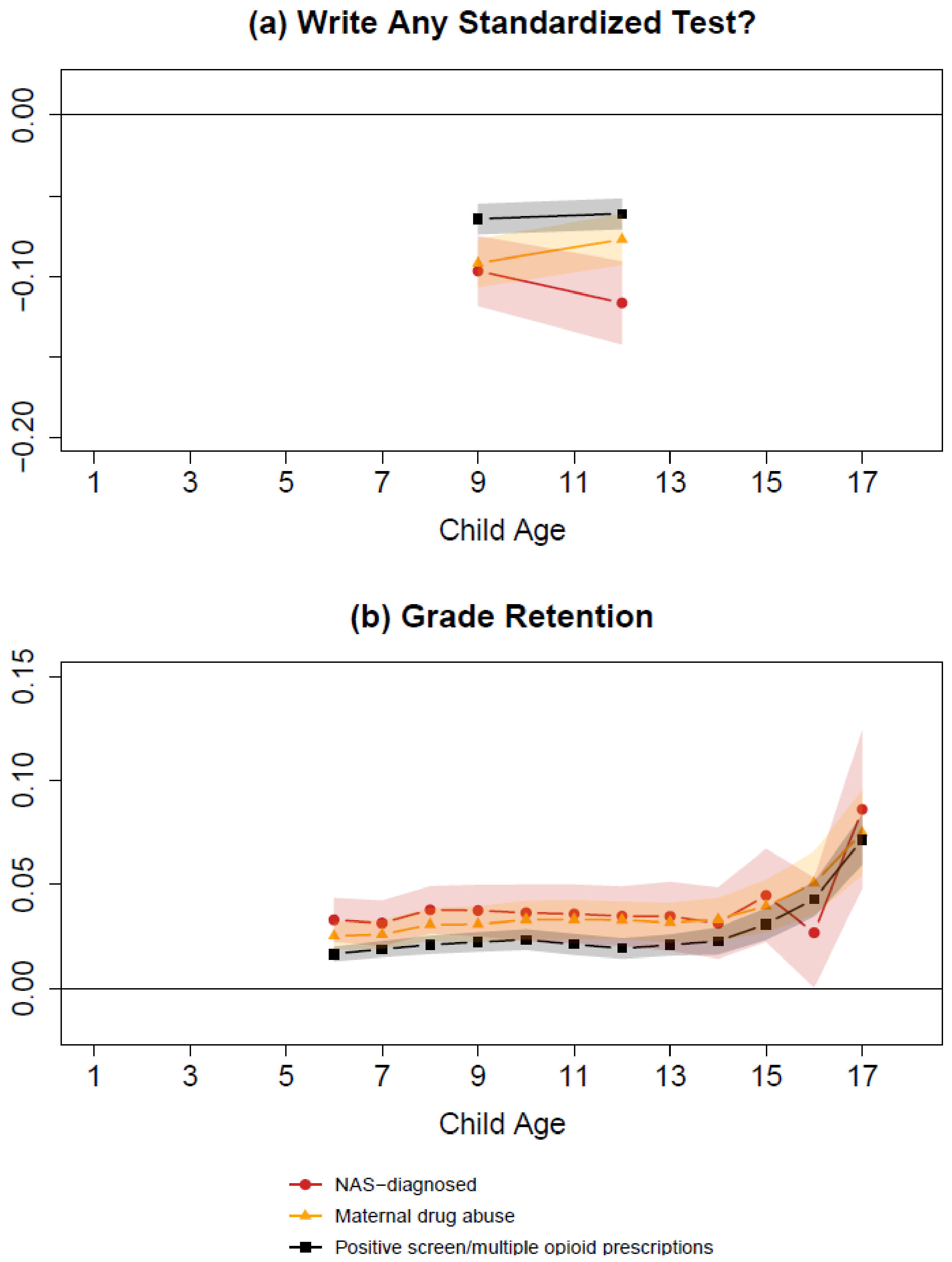

Each point is an estimated coefficient on the exposure measure (representing the difference between exposed vs non-exposed), along with its 95% confidence interval, from equation 1: linear regression comparing the stated outcome variable between the exposed and those never exposed, controlling for fixed effects for birth year, birth month, mother's age at birth, parity, 3-digit postal code, calendar year, mother's world region of birth, marital status, Indigenous identity, an indicator for receiving government benefit during pregnancy, and categorical bins for low income proxy. Means for non-exposed children for writing any standardized test is 0.57 and grade retention is 0.028 in ages 6-12 and 0.062 in ages 13-18.

Supplemental Figure 4. Covariate balance in propensity score matched sample

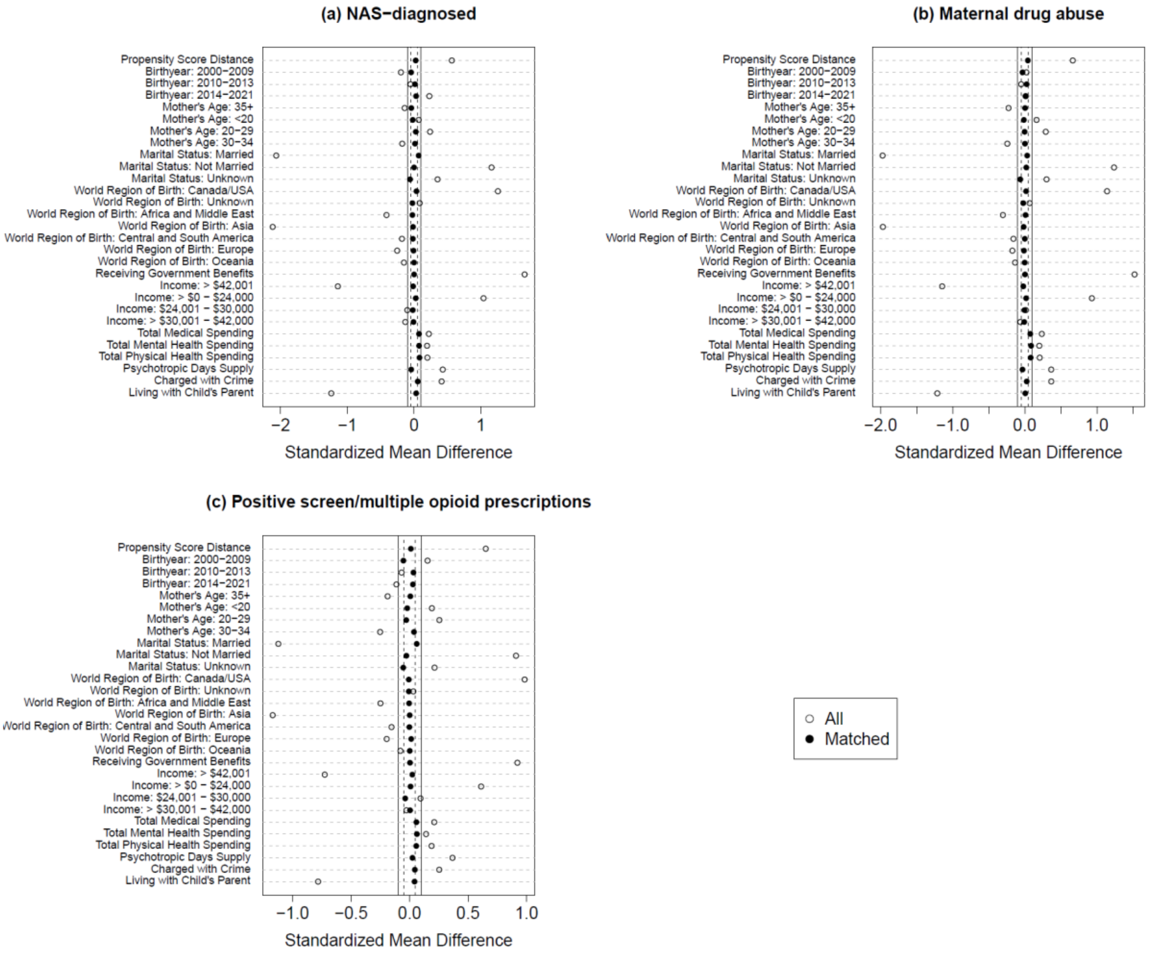

This figure shows covariate balance before and after propensity score matching for the three exposure measures (in panels a-c). All covariates used in the propensity score matching are displayed, as well as the propensity score distance. All covariates are maternal covariates, measured in the year prior to childbirth. Standardized mean difference (SMD) for each covariate is defined as the difference in means between the exposed and non-exposed group, divided by the pooled standard deviation of the two. Hollow points correspond to the SMD for all (unmatched) children, which is the sample used in our baseline analyses. Solid points correspond to the SMD for (propensity score) matched children. Solid and dashed vertical lines correspond to 0.1 and 0.05 SMD, respectively. See the Supplemental Information for more details on the propensity score matching procedure.

Supplemental Table 1. Regression output for healthcare utilization outcomes (Figure 1)

	Total Medical Spending			Total Drug Spending		
	1	2	3	1	2	3
**1**	376.4 (64.7)	207.4 (39.4)	199.9 (24.1)	34.7 (5.9)	19.5 (3.1)	17.4 (1.6)
**2**	121.9 (41)	85.9 (28.2)	93.1 (14.3)	29.8 (5.8)	13.7 (3.3)	15 (1.9)
**3**	123.5 (37.7)	48.3 (18.2)	88.9 (11.8)	29.1 (7)	14.8 (2.8)	13.4 (2)
**4**	86.1 (26.4)	93.4 (21.8)	73.1 (12.2)	29.3 (7.2)	12.8 (3.3)	14.8 (2.3)
**5**	129 (36.3)	101.3 (23.3)	81.8 (13.4)	32.3 (9.8)	19.4 (4.5)	16.5 (2.5)
**6**	106.6 (35.8)	73.2 (19.1)	65.5 (12.1)	50 (9.2)	33.1 (5.4)	25.1 (2.8)
**7**	170.9 (46.2)	102.8 (23.5)	85.8 (12.6)	88.8 (13.8)	57.1 (7.2)	39.4 (3.6)
**8**	136.7 (45.7)	98.4 (25.9)	96.5 (12.9)	116.6 (15.9)	76.8 (8.4)	54.2 (3.8)
**9**	135.5 (50)	162.4 (32.5)	92 (13.8)	148 (20)	94.9 (10.4)	62.5 (4.8)
**10**	148.8 (52.6)	85.5 (27.1)	80 (16.8)	165.4 (23.1)	107.5 (11.9)	74.4 (6)
**11**	171.4 (61.7)	110.6 (33.8)	90.9 (18.8)	161.3 (22.1)	117.2 (13)	75.5 (6.2)
**12**	113.3 (56.9)	127.2 (37.4)	74.6 (18.7)	161.5 (23.9)	121.1 (14.4)	77.8 (6.3)
**13**	238.2 (89.5)	132.3 (43.7)	133 (24.4)	171.5 (27.7)	134.6 (15.4)	79 (6.7)
**14**	89.1 (85.4)	185.2 (59.3)	140.2 (24.9)	162.9 (29.9)	125.8 (16.2)	75.5 (7.2)
**15**	395.1 (132.5)	335.8 (70.9)	172.1 (33.7)	180.3 (35.3)	117.1 (17.3)	81.6 (7.6)
**16**	247.3 (139.1)	246 (82.7)	229 (44)	168.6 (33)	108.7 (17.8)	83 (8.7)
**17**	251.4 (161.6)	172.5 (84.8)	202.8 (48.8)	148.7 (43.8)	95.1 (18.8)	81.4 (10)
**18**	354 (184.9)	180.1 (80.6)	178.6 (48.3)	197.8 (55.8)	89.5 (22.6)	75.3 (12.9)

Each cell displays the estimated regression coefficient on exposure from Equation 1, as well as its standard error in parentheses. The rows correspond to each age. Measures 1, 2, and 3 correspond to "NAS-diagnosed", "Maternal drug abuse", and "Positive screen/multiple opioid prescriptions", respectively.

Supplemental Table 2. Regression output for select inclusive education designations (Figure 2).

	Any Inclusive Education			Physical Dsblty/Chronic Impair			Mental Illness; Require Behav. Support/Interv.			Intellectual Disability; ASD			Learning Disability		
	1	2	3	1	2	3	1	2	3	1	2	3	1	2	3
**6**	0.113 (0.013)	0.086 (0.009)	0.048 (0.004)	0.076 (0.01)	0.059 (0.007)	0.026 (0.003)	0.03 (0.006)	0.022 (0.003)	0.016 (0.002)	0.008 (0.005)	0.003 (0.003)	0.004 (0.002)	0 (0.001)	0.001 (0.001)	0 (0)
7	0.144 (0.014)	0.111 (0.01)	0.062 (0.004)	0.096 (0.011)	0.079 (0.008)	0.035 (0.004)	0.035 (0.007)	0.028 (0.004)	0.022 (0.002)	0.01 (0.005)	0.003 (0.003)	0.005 (0.002)	0.002 (0.002)	0.001 (0.001)	0 (0.001)
8	0.165 (0.015)	0.124 (0.011)	0.072 (0.005)	0.121 (0.012)	0.096 (0.01)	0.044 (0.004)	0.028 (0.009)	0.025 (0.004)	0.022 (0.002)	0.011 (0.006)	0.003 (0.003)	0.006 (0.002)	0.003 (0.004)	0 (0.002)	0 (0.001)
**9**	0.162 (0.014)	0.124 (0.011)	0.079 (0.005)	0.137 (0.014)	0.111 (0.01)	0.052 (0.005)	0.016 (0.007)	0.017 (0.004)	0.019 (0.002)	0.008 (0.006)	0.001 (0.003)	0.006 (0.002)	0.001 (0.005)	-0.004 (0.003)	0.002 (0.002)
10	0.162 (0.016)	0.127 (0.012)	0.085 (0.006)	0.145 (0.015)	0.12 (0.011)	0.06 (0.005)	0.013 (0.01)	0.013 (0.005)	0.018 (0.003)	0.006 (0.007)	-0.001 (0.003)	0.004 (0.002)	-0.001 (0.007)	-0.004 (0.004)	0.004 (0.002)
11	0.171 (0.016)	0.13 (0.011)	0.09 (0.006)	0.166 (0.016)	0.128 (0.011)	0.065 (0.005)	0.007 (0.01)	0.014 (0.005)	0.017 (0.003)	0.004 (0.007)	-0.006 (0.003)	0.004 (0.002)	-0.007 (0.008)	-0.005 (0.005)	0.006 (0.003)
12	0.173 (0.015)	0.125 (0.01)	0.093 (0.006)	0.177 (0.016)	0.136 (0.011)	0.067 (0.005)	0.003 (0.009)	0.012 (0.005)	0.017 (0.003)	0.002 (0.007)	-0.011 (0.003)	0.003 (0.002)	-0.009 (0.009)	-0.011 (0.006)	0.006 (0.003)
13	0.182 (0.016)	0.128 (0.012)	0.097 (0.006)	0.187 (0.016)	0.136 (0.012)	0.069 (0.005)	0 (0.009)	0.014 (0.005)	0.02 (0.003)	0.003 (0.008)	-0.009 (0.004)	0.003 (0.003)	-0.008 (0.011)	-0.013 (0.005)	0.007 (0.003)
14	0.186 (0.019)	0.123 (0.013)	0.100 (0.007)	0.171 (0.019)	0.128 (0.012)	0.068 (0.005)	0.012 (0.012)	0.018 (0.006)	0.023 (0.003)	0.004 (0.009)	-0.008 (0.004)	0.003 (0.003)	-0.002 (0.012)	-0.014 (0.006)	0.007 (0.004)
15	0.200 (0.02)	0.13 (0.014)	0.108 (0.007)	0.189 (0.02)	0.133 (0.013)	0.072 (0.006)	0.004 (0.012)	0.018 (0.007)	0.028 (0.004)	0.007 (0.01)	-0.007 (0.004)	0.003 (0.003)	0 (0.012)	-0.012 (0.008)	0.005 (0.004)
16	0.182 (0.026)	0.132 (0.015)	0.106 (0.008)	0.167 (0.022)	0.133 (0.014)	0.068 (0.006)	0.019 (0.016)	0.024 (0.008)	0.027 (0.005)	-0.005 (0.009)	-0.012 (0.005)	0.003 (0.004)	0.005 (0.013)	-0.012 (0.007)	0.009 (0.005)
17	0.178 (0.029)	0.125 (0.015)	0.097 (0.008)	0.16 (0.024)	0.121 (0.013)	0.065 (0.005)	0.022 (0.02)	0.03 (0.008)	0.028 (0.006)	-0.002 (0.012)	-0.009 (0.006)	0.005 (0.005)	0.001 (0.015)	-0.015 (0.008)	0 (0.005)
18	0.153 (0.037)	0.135 (0.023)	0.1 (0.016)	0.167 (0.037)	0.158 (0.023)	0.092 (0.012)	0.013 (0.029)	0.007 (0.019)	0.014 (0.012)	0.012 (0.028)	-0.013 (0.014)	0.006 (0.008)	-0.036 (0.018)	-0.014 (0.012)	-0.01 (0.008)

Each cell displays the estimated regression coefficient on exposure from Equation 1, as well as its standard error in parentheses. The rows correspond to each age. Measures 1, 2, and 3 correspond to “NAS-diagnosed”, “Maternal drug abuse”, and “Positive screen/multiple opioid prescriptions”, respectively.

Supplemental Table 3. Regression output for educational outcomes (Figure 3).

	Standardized Math			Standardized Reading			Standardized Writing			Graduate High School			High School GPA		
	1	2	3	1	2	3	1	2	3	1	2	3	1	2	3
9	-0.084 (0.031)	-0.041 (0.025)	-0.088 (0.012)	-0.019 (0.039)	0.015 (0.026)	-0.054 (0.013)	-0.081 (0.034)	-0.024 (0.026)	-0.065 (0.013)						
12	-0.142 (0.035)	-0.09 (0.027)	-0.118 (0.013)	-0.102 (0.041)	-0.022 (0.027)	-0.079 (0.014)	-0.127 (0.037)	-0.057 (0.028)	-0.078 (0.013)						
18										-0.1 (0.023)	-0.103 (0.013)	-0.085 (0.007)	-0.155 (0.077)	-0.124 (0.043)	-0.139 (0.026)

Each cell displays the estimated regression coefficient on exposure from Equation 1, as well as its standard error in parentheses. The rows correspond to each age. Measures 1, 2, and 3 correspond to “NAS-diagnosed”, “Maternal drug abuse”, and “Positive screen/multiple opioid prescriptions”, respectively.

Supplemental Table 4. Regression output for government welfare utilization and child welfare outcomes (Figure 4).

	Monthly Government Transfers			Any Income Assistance			Any Disability Assistance			Under Supervision of Child Protective Services			Foster Care		
	1	2	3	1	2	3	1	2	3	1	2	3	1	2	3
1	334 (15.4)	313 (10)	172.5 (6.2)	0.124 (0.015)	0.127 (0.013)	0.098 (0.005)	0.183 (0.012)	0.16 (0.012)	0.071 (0.006)	0.416 (0.016)	0.313 (0.015)	0.142 (0.008)	0.295 (0.015)	0.207 (0.011)	0.093 (0.006)
2	361.3 (16.6)	334.1 (11.4)	188 (6.8)	0.129 (0.015)	0.12 (0.013)	0.098 (0.005)	0.19 (0.013)	0.172 (0.013)	0.08 (0.006)	0.331 (0.013)	0.257 (0.011)	0.124 (0.007)	0.221 (0.011)	0.166 (0.01)	0.077 (0.005)
3	380 (17.3)	345.2 (12.6)	193.2 (7.2)	0.136 (0.013)	0.118 (0.011)	0.097 (0.005)	0.191 (0.012)	0.178 (0.012)	0.086 (0.006)	0.239 (0.013)	0.2 (0.01)	0.102 (0.006)	0.149 (0.01)	0.128 (0.008)	0.063 (0.005)
4	388.4 (17.8)	350.5 (13.2)	201.4 (7.9)	0.125 (0.012)	0.115 (0.011)	0.095 (0.005)	0.203 (0.013)	0.184 (0.012)	0.093 (0.006)	0.186 (0.012)	0.168 (0.009)	0.089 (0.005)	0.117 (0.008)	0.106 (0.007)	0.053 (0.004)
5	381.4 (17.6)	348.5 (12.4)	204.8 (7.9)	0.117 (0.01)	0.103 (0.01)	0.088 (0.005)	0.209 (0.011)	0.19 (0.011)	0.098 (0.006)	0.151 (0.01)	0.147 (0.008)	0.078 (0.004)	0.105 (0.008)	0.097 (0.007)	0.048 (0.003)
6	403.6 (17.3)	348.9 (12.6)	205.5 (7.7)	0.127 (0.011)	0.099 (0.008)	0.082 (0.004)	0.218 (0.012)	0.196 (0.011)	0.103 (0.005)	0.127 (0.009)	0.127 (0.008)	0.071 (0.004)	0.091 (0.007)	0.089 (0.007)	0.044 (0.003)
7	416.3 (17.5)	348.3 (12.3)	210.2 (7.3)	0.131 (0.011)	0.097 (0.008)	0.082 (0.004)	0.211 (0.012)	0.193 (0.01)	0.106 (0.005)	0.114 (0.01)	0.119 (0.008)	0.068 (0.005)	0.09 (0.008)	0.083 (0.007)	0.042 (0.004)
8	419.8 (19.3)	351.1 (13.5)	213.5 (7.8)	0.124 (0.011)	0.09 (0.008)	0.076 (0.004)	0.224 (0.012)	0.202 (0.011)	0.111 (0.005)	0.119 (0.012)	0.115 (0.008)	0.065 (0.004)	0.087 (0.01)	0.081 (0.007)	0.041 (0.003)
9	438 (23.1)	356.3 (13.6)	215.6 (7.5)	0.107 (0.011)	0.089 (0.008)	0.072 (0.004)	0.235 (0.014)	0.203 (0.01)	0.115 (0.005)	0.115 (0.012)	0.108 (0.008)	0.062 (0.004)	0.081 (0.011)	0.076 (0.007)	0.04 (0.003)
10	430 (23.7)	354.2 (13.6)	223.4 (7.5)	0.1 (0.012)	0.071 (0.008)	0.069 (0.004)	0.244 (0.014)	0.214 (0.01)	0.123 (0.006)	0.115 (0.011)	0.096 (0.008)	0.06 (0.004)	0.08 (0.01)	0.067 (0.007)	0.037 (0.003)
11	416.5 (26.7)	353 (14.6)	226.9 (8.4)	0.075 (0.013)	0.065 (0.008)	0.058 (0.004)	0.256 (0.017)	0.221 (0.01)	0.132 (0.006)	0.106 (0.01)	0.089 (0.008)	0.057 (0.004)	0.08 (0.01)	0.064 (0.007)	0.034 (0.003)
12	411.5 (27)	345.7 (14.5)	230.7 (8.3)	0.083 (0.014)	0.07 (0.009)	0.057 (0.004)	0.252 (0.018)	0.217 (0.011)	0.134 (0.006)	0.119 (0.011)	0.088 (0.007)	0.055 (0.004)	0.089 (0.011)	0.066 (0.007)	0.035 (0.003)
13	417.3 (27.4)	347.9 (14.3)	230.5 (8.6)	0.076 (0.014)	0.063 (0.007)	0.048 (0.004)	0.265 (0.018)	0.219 (0.01)	0.135 (0.006)	0.118 (0.013)	0.088 (0.008)	0.052 (0.004)	0.083 (0.011)	0.058 (0.007)	0.034 (0.003)
14	405.7 (28.9)	330.3 (14.8)	223.7 (8.6)	0.077 (0.013)	0.056 (0.008)	0.045 (0.004)	0.255 (0.02)	0.213 (0.011)	0.132 (0.006)	0.115 (0.012)	0.085 (0.008)	0.053 (0.004)	0.079 (0.011)	0.056 (0.007)	0.032 (0.003)
15	428.5 (31.8)	338.2 (18.4)	228.7 (9.7)	0.078 (0.015)	0.059 (0.008)	0.044 (0.004)	0.264 (0.021)	0.211 (0.012)	0.131 (0.006)	0.11 (0.013)	0.087 (0.009)	0.055 (0.005)	0.071 (0.01)	0.059 (0.008)	0.032 (0.003)
16	481.5 (35.9)	338 (20.3)	229.8 (11.2)	0.071 (0.014)	0.047 (0.01)	0.039 (0.004)	0.275 (0.023)	0.212 (0.013)	0.135 (0.007)	0.122 (0.014)	0.092 (0.01)	0.057 (0.005)	0.076 (0.011)	0.058 (0.008)	0.03 (0.004)
17	469.6 (38.5)	327.2 (19.6)	229.6 (12.4)	0.058 (0.016)	0.038 (0.009)	0.032 (0.005)	0.27 (0.025)	0.204 (0.012)	0.134 (0.008)	0.111 (0.016)	0.089 (0.01)	0.064 (0.005)	0.062 (0.011)	0.066 (0.009)	0.03 (0.004)
18	481.5 (47.6)	329.4 (22.7)	238 (14.2)	0.046 (0.016)	0.04 (0.009)	0.034 (0.005)	0.29 (0.026)	0.198 (0.014)	0.137 (0.009)	0.416 (0.016)	0.313 (0.015)	0.142 (0.008)	0.295 (0.015)	0.207 (0.011)	0.093 (0.006)

Each cell displays the estimated regression coefficient on exposure from Equation 1, as well as its standard error in parentheses. The rows correspond to each age. Measures 1, 2, and 3 correspond to “NAS-diagnosed”, “Maternal drug abuse”, and “Positive screen/multiple opioid prescriptions”, respectively.

Supplemental Table 5. Regression output for cumulative mortality (Figure 5).

	Cumulative Mortality		
	1	2	3
1	0.0016 (0.0017)	0.0024 (0.0011)	0.0018 (5e-04)
2	0.0018 (0.0017)	0.0026 (0.0011)	0.002 (5e-04)
3	0.0016 (0.0017)	0.0025 (0.0011)	0.0019 (5e-04)
4	0.0025 (0.0019)	0.0025 (0.0011)	0.0017 (6e-04)
5	0.0038 (0.002)	0.0034 (0.0012)	0.002 (6e-04)
6	0.0038 (0.0022)	0.0036 (0.0013)	0.002 (6e-04)
7	0.0038 (0.0023)	0.0037 (0.0015)	0.002 (6e-04)
8	0.0025 (0.0023)	0.0038 (0.0015)	0.002 (6e-04)
9	0.0023 (0.0023)	0.004 (0.0016)	0.0022 (7e-04)
10	0.0021 (0.0023)	0.0037 (0.0017)	0.0023 (7e-04)
11	0.0024 (0.0025)	0.0039 (0.0017)	0.0026 (7e-04)
12	0.0029 (0.0027)	0.0036 (0.0017)	0.0028 (7e-04)
13	0.0021 (0.0028)	0.0042 (0.0017)	0.0037 (8e-04)
14	8e-04 (0.0024)	0.0036 (0.0017)	0.0035 (9e-04)
15	5e-04 (0.0026)	0.004 (0.0018)	0.0039 (0.001)
16	-0.0013 (0.0026)	0.0036 (0.002)	0.0038 (0.001)
17	-8e-04 (0.003)	0.0046 (0.0023)	0.0045 (0.0012)
18	0.0018 (0.0046)	0.0057 (0.0028)	0.0041 (0.0013)

Each cell displays the estimated regression coefficient on exposure from Equation 1, as well as its standard error in parentheses. The rows correspond to each age. Measures 1, 2, and 3 correspond to "NAS-diagnosed", "Maternal drug abuse", and "Positive screen/multiple opioid prescriptions", respectively.

B. Definitions

Defining In Utero Exposure Measures

Measure 1 (“NAS-diagnosed”):

At the childbirth delivery episode
ICD-10: P96.1
ICD-9: 779.5

Measure 2 (“Maternal drug abuse”):

Newborn diagnosed with NAS-related diagnosis codes in an inpatient hospital setting:
ICD-10: P96.1, P.044, P.041
ICD-9: 779.5, 760.70, 760.72, 760.73, 760.75, 760.79

Mothers diagnosed with drug abuse, drug dependence during pregnancy (e.g., known from gestation age of newborn) and at childbirth delivery:
ICD-10: F11, F13, F14, F15, F16, T39, T40, T41, T43.6
ICD-9: 305.5, 965.0, 970.1, 965, 967, 970, E935.0, E935.1, E935.2, E940.1, E850, E852, E855, 304 and not 304.3

Measure 3 (“Positive screen/multiple opioid prescriptions”):

Drug use during pregnancy identified as a risk via clinical screen: heroin, cocaine, prescription drugs, and methadone

Three or more separate opioid prescriptions (not counting refills) during pregnancy period. Opioids do not include methadone, buprenorphine.

Maternal Variable Definitions

Mother’s age bins: under 20; 20-24; 25-29; 30-34; 35-39; 40 and higher

Parity bins: 1, 2, and 3 or more

Variable for Indigenous identity comes from child’s school enrollment records (self-reported Indigenous ancestry) as well as various group identifiers for band-affiliated MSP (health insurance) plans.

Mother’s world region of birth is a categorical variable for Canada/USA, Asia, Europe, Africa and Middle East, South America, and Oceania.

Mother’s marital status is from the birth record.

Mother’s government benefits include disability, welfare benefits, and pharmacare or medical premium subsidies.

Mother's low income bins are proxied from medical premium subsidies which provide tiered subsidies for the following household income levels (in Canadian Dollars): \$0 - \$24,000; \$24,001 - \$30,000; \$30,000-\$42,000.

Health Data

We observe all payments made to physicians under the provincial universal health insurance program, the Medical Services Plan (MSP). We also observe all inpatient and day surgery visits in hospitals via the Discharge Abstract Database (DAD). Each observation in the DAD records a Resource Intensity Weight (RIW) based on the patient's case mix. The RIW is multiplied by a "Cost per Weighted Case", or CPWC, to estimate that visit's cost of care. The sum of RIW×CPWC within the province equates to total hospital expenditure, although RIW×CPWC may over- or under-estimate the cost for any given visit. To calculate a person's total healthcare expenditure, we sum MSP physician billings and hospital RIW×CPWC incurred during the year.

The main healthcare exclusions from the combined MSP and DAD data are non-medically necessary dental, vision, and allied health services (e.g., massage therapy). Emergency Department (ED) visits are separately tracked in the National Ambulatory Care Reporting System, but tracking in British Columbia was poor before 2014. Nonetheless, approximately 70% of emergency room visits can be identified through a combination of the MSP and DAD data. Specifically, any ED visit that either (i) resulted in an inpatient admission or (ii) had a physician perform a procedure in the ED that was billed through the MSP. We use this as our measure of ED visits for 2000-2020.

Pharmanet is the provincial tracking system for prescriptions filled in community pharmacies. It includes the prescription cost and the AHFS Pharmacologic-Therapeutic Classification code. Most pharmaceuticals are filled through community pharmacists. Pharmaceutical treatments in hospital settings are incorporated into the estimated cost of an inpatient visit and not tracked by Pharmanet.

C. Propensity Score Matching

We implement one-to-one nearest-neighbor propensity score matching. Specifically, in the first step, we estimate propensity scores via a logistic regression model. For each of our three exposure measures, we estimate for untreated (non-exposed in any form) observations the probability of being exposed using the following covariates: birth year periods, mother age bins, marital status on birth certificate, mother's place of birth, indicator for mother receiving government benefits in the year prior to birth, and proxies for low income), as well as prior calendar year (relative to calendar year of birth) total medical spending, total mental health medical spending, total physical health medication spending, number of days supply of psychotropics, indicator for being charged with a crime, and proxy for parents living together (via paying for health insurance premiums together). Then one-to-one nearest-neighbor

matching without replacement is used to match each exposed newborn with the most similar untreated observation based on the estimated propensity score, thereby improving covariate balance between exposed and non-exposed children. This was implementing using the R package MatchIt.
Due to our large sample of births, all "treated" observations in each of our three exposed measures were matched to an "untreated" observation. In other words, the sample size of the exposed and non-exposed children were the same, in each of the three comparisons. Then we estimated least squares regressions comparing exposed to matched non-exposed children (analogous to our baseline empirical specification), controlling for 3-digit postal code.

D. Calculation of the Number of Potentially In Utero Exposed Newborns

In our British Columbia (BC) sample, the rate of in utero exposed newborns—excluding those flagged only by opioid prescription data to be conservative—is 21.1 per 1,000 live births, compared to a 4.9 per 1,000 rate for neonatal abstinence syndrome (NAS). Given that there were 41,403 births in BC in 2023, this translates to approximately 874 potentially exposed newborns that year. To estimate comparable figures for the United States, we assume the same undercount ratio observed in BC. Since the U.S. reports a NAS rate of 6 per 1,000 births, applying BC's ratio implies 25.8 in utero exposed infants per 1,000 births in the U.S. With 3,667,758 births recorded in 2022, this yields an estimated 94,762 U.S. newborns potentially exposed to opioids each year. This is comparable to the number of drug overdose deaths in the U.S. in 2022: 107,941.
